# CO_2_ as an Alternative to Traditional Antiscalants in Pressure-Driven Membrane Processes: An Experimental Study of Lab-Scale Operation and Cleaning Strategies

**DOI:** 10.3390/membranes12100918

**Published:** 2022-09-22

**Authors:** Muhammad Kashif Shahid, Younggyun Choi

**Affiliations:** 1 Research Institute of Environment & Biosystem, Chungnam National University, Daejeon 34134, Korea; 2 Department of Environmental & IT Engineering, Chungnam National University, Daejeon 34134, Korea

**Keywords:** CO_2_ utilization, fouling, membrane, pollution, wastewater treatment, circular economy

## Abstract

Scaling, or inorganic fouling, is a major factor limiting the performance of membrane-based water treatment processes in long-term operation. Over the past few decades, extensive studies have been conducted to control the scale growth found in membrane processes and to develop sustainable and greener processes. This study details the role of CO_2_ in scale inhibition in membrane processes. The core concept of CO_2_ utilization is to reduce the influent pH and to minimize the risk of scale formation from magnesium or calcium salts. Three reverse osmosis (RO) units were operated with a control (U1), CO_2_ (U2), and a commercial antiscalant, MDC-220 (U3). The performances of all the units were compared in terms of change in transmembrane pressure (TMP). The overall efficiency trend was found as U1 > U3 > U2. The membrane surfaces were analyzed using Scanning Electron Microscopy (SEM) and Energy Dispersive Spectroscopy (EDS) for the morphological and elemental compositions, respectively. The surface analysis signified a significant increase in surface smoothness after scale deposition. The noticeable reduction in surface roughness can be described as a result of ionic deposition in the valley region. A sludge-like scale layer was found on the surface of the control membrane (U1) which could not be removed, even after an hour of chemical cleaning. After 20–30 min of cleaning, the U2 membrane was successfully restored to its original state. In brief, this study highlights the sustainable membrane process developed via CO_2_ utilization for scale inhibition, and the appropriate cleaning approaches.

## 1. Introduction

Global population growth and uncontrolled water and energy consumption have resulted in serious environmental and health consequences [1]. The population of the world is rising by ca. 80 million/annum and is estimated to cross 9 billion by 2050. This will drastically increase the global energy demand, necessitating 70% more food, 50% more fresh water, and 50% more fuel [2]. To meet water demand, water reclamation, desalination, and reuse are common practices used at present [3]. Water reclamation is considered one of the major resources for generating improved-quality water for industrial application, such as for low-pressure boiler make-up water, cooling water in recirculating systems, and ultrapure water for semiconductor manufacturing [4]. Reverse osmosis (RO) is a widely applied technology for pure water production from reclaimed water [5,6]. However, the scale formation found in membrane processes is a challenging issue for water professionals [7,8].

Scaling, or inorganic fouling, refers to the nucleation, crystallization, and gathering of mineral deposits on a membrane surface [9]. Once the concentration of sparingly soluble salts crosses their solubility limits, precipitation happens. Most natural water sources have scale-forming ions, which have a tendency for precipitation. The ionic composition and concentration always vary among water sources due to the diversity of hydrogeological environments, such as site, climate, season, and type of source water (e.g., surface water, groundwater, sea water, and wastewater). The common scale may contain a single salt, or a mixture of many salts, such as calcium sulfate (CaSO_4_), calcium carbonate (CaCO_3_), magnesium hydroxide (Mg(OH)_2_), strontium sulfate (SrSO_4_), barium sulfate (BaSO_4_), and silica (SiO_2_) [10].

Scale deposition deteriorates the operational efficiency of membrane-based water treatment processes, if not addressed in a timely manner. Following scale development, an increase in salt permeation, a decrease in permeate flux, or a rise in transmembrane pressure (TMP) may result in the production of low-quality product water and higher operational and maintenance costs [11]. Membrane-based water and wastewater treatment processes generally utilize chemical additives or antifouling agents to minimize the scale-forming tendency of feed water [12,13]. An antifouling agent’s function is to delay the onset of crystallization, therefore raising the induction time required for crystallization [5,14]. The acidification of feed water, for instance, through the addition of sulfuric acid or hydrochloric acid into feed water, is another scale inhibition approach that raises the solubility of sparingly soluble salts by reducing the influent pH [5].

Earlier studies have presented the application of air micro–nano bubbles as a greener approach to improve the cleaning efficiency of RO processes [8]. The micro–nano bubbles were applied to agitate and detach the foulants from the surface of the membrane. Although this approach appeared to be effective for cleaning the fouled membrane, the method can only be applied to enhance the efficiency of clean-in-place (CIP) procedures during RO operations and cannot be used as an alternative for antiscalants. Moreover, the operation of the bubble generator and the control measures for bubble size distribution may lead to a significant rise in the energy consumption and operational cost of the RO system. Another study applied a CO_2_-saturated solution to clean the biofilm and particulate fouling from the spacer channels of the RO membranes [15]. The CO_2_ nucleation indicated a higher cleaning efficiency when compared with N_2_ sparging and water rinsing. In short, the air micro–nano bubbles and dissolved CO_2_ solutions were found to be highly effective for removing the biofilm and particulate foulants from the membrane; however, their role in the inhibition of scale formation in membrane-based water treatment systems was not examined.

In our earlier study, we applied CO_2_ as a green approach to control the fouling in reverse osmosis operation during wastewater reclamation [16]. We examined the efficiency of RO units in a constant pressure mode of operation and found impressive results. The main purpose of using CO_2_ in RO processes is to reduce the influent pH and to minimize the risk of scale formation from magnesium or calcium salts. The main objectives of this study are to examine (a) the operation of RO units with a constant flux mode of operation, and (b) the practical applicability of different cleaning strategies to restore the initial state of the fouled membranes. Three RO units were operated with a control, CO_2_, and an antiscalant, respectively. A detailed membrane autopsy was conducted to identify the nature and extent of scale deposition on the surface of the membranes. Moreover, different cleaning protocols (CP) were adopted to determine the best-suited sustainable CP for fouled membranes. The outcomes of this study signify the practical application of the proposed green scale-inhibition approach to membrane-based water and wastewater treatment systems.

## 2. Materials and Methods

### 2.1. Membrane and Chemical Agents

Analytical research-grade reagents and chemicals were obtained from Fisher Scientific and utilized as received. The synthetic wastewater was used as feed for RO units. Considering the potential application of this study in advanced wastewater reclamation processes, the feed water quality was maintained in line with the quality of tertiary-treated effluents of domestic wastewater treatment plants. The wastewater was prepared using reagent-grade chemicals. The molar concentration of the chemicals added into the feed water included 0.07 mM of KH_2_PO_4_, 0.08 mM of MgSO_4_, 0.30 mM of KNO_3_, 1.14 mM of KCl, 3.4 mM of MgCl_2_, 5 mM of CaCl_2_, and 20 mM of NaCl. Hypersperse™ MDC 220, a commercial antifouling agent, was acquired from GE Company, Shanghai, China. It is worth mentioning that MDC 220 is a widely applied antiscalant in wastewater reclamation plants in South Korea. It contains phosphonic acid, (1-hydroxyethylidene) bis-sodium salt (C_2_H_7_NaO_7_P_2_). It has specific gravity of 1.169, and is completely soluble in water. It is suggested for all kinds of RO membrane by manufacturer (GE Company). The RO membrane filters were purchased from HYUNDAI Wacortec, Seoul, South Korea, and the membrane specifications are described in Table 1.

### 2.2. Operation Strategy of the RO Systems

**Figure 1:**
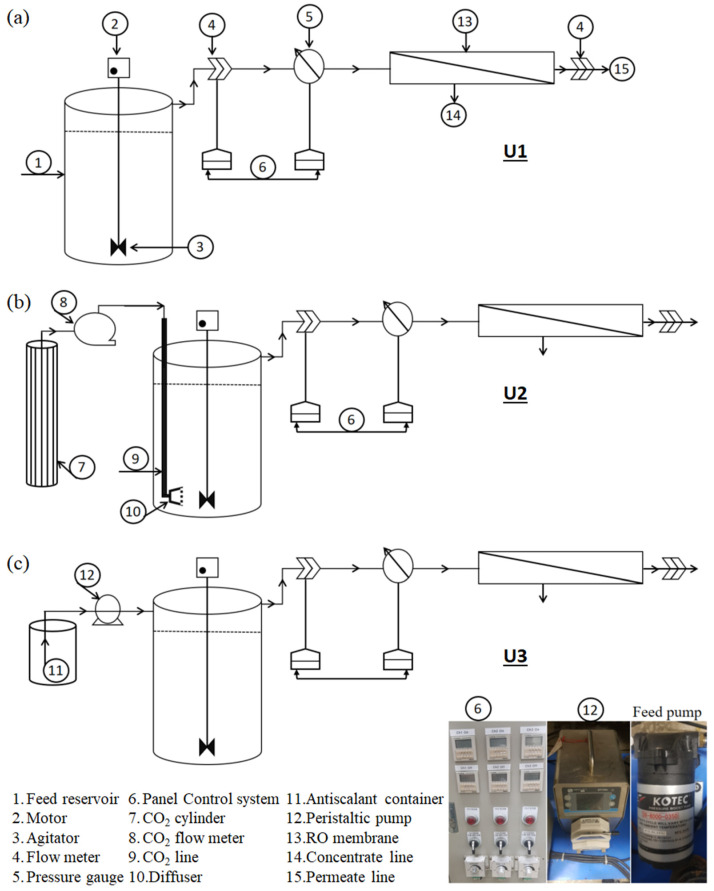
Schematic illustration of (**a**) U1, (**b**) U2, and (**c**) U3.

The RO membrane filters were rinsed prior to being applied to wastewater filtration. Three single-pass, stand-alone RO units were operated with a control, CO_2_, and an antiscalant, and categorized as U1, U2, and U3, respectively. The influent flow rate and permeate flux were maintained at 40 L/day and 2.32 LMH, respectively. The recovery rate was adjusted to 56% and the RO units were operated at 2 bar initial TMP. The influent pH was 7.00 ± 0.10 for U1 and U3, and was reduced to 6 by the addition of CO_2_ in U2. The CO_2_ injection rate was 150 mL/min, and nearly 1 min injection time was consumed to reach the desired pH level. Once the pH of the feed water was stabilized, U3 was operated. Figure 1 highlights the scheme of RO units operated in this study. Following filtration, the fouled membranes were cleaned using 1% EDTA (ethylenediaminetetraacetic acid), 0.2% HCl, and 0.1% sodium hydroxide solutions. The membrane cleaning was performed for different durations, ranging from 10 to 60 min, and the effect of contact time on cleaning efficiency was determined. The operational variables such as change in TMP, recovery %, pH, conductivity, and concentration of particular ions in the influent, permeate, and concentrate streams were constantly monitored during operation.

A membrane autopsy was conducted to identify the nature of fouling during the filtration process. The membrane element was instantly opened and prepared for examination after the RO unit was shut down. The membrane element was removed from the pressure vessel (membrane housing) and then both surfaces of each leaf of the membrane module were carefully examined for any physical damage. Later, several small membrane pieces were cut from various sheets, and the fouling layer was examined with advanced instrumental techniques.

### 2.3. Instrumentation

The pH and conductivity were analyzed using a 96pH-L2 (Samsan, Korea) and an EC96 (M-Cubic Co., Ltd., Yuseong-gu, Daejeon, Korea), respectively. The concentrations of cations and anions in the water samples were determined with Thermo Scientific™ Dionex™ ICS-1000 and Thermo Scientific™ Dionex™ ICS-5000 (Carlsbad, CA, USA), respectively. The surface characterization of the RO membranes was performed using Scanning Electron Microscopy (SEM, Hitachi SU-70, Tokyo, Japan) coupled with Energy Dispersive Spectroscopy (EDS), manufactured by Hitachi Ltd., Tokyo, Japan. The surface-roughness statistics of the virgin and fouled membranes were determined by considering the topographical images taken using SEM analysis.

## 3. Results and Discussion

### 3.1. Operational Performances of RO Units

**Figure 2:**
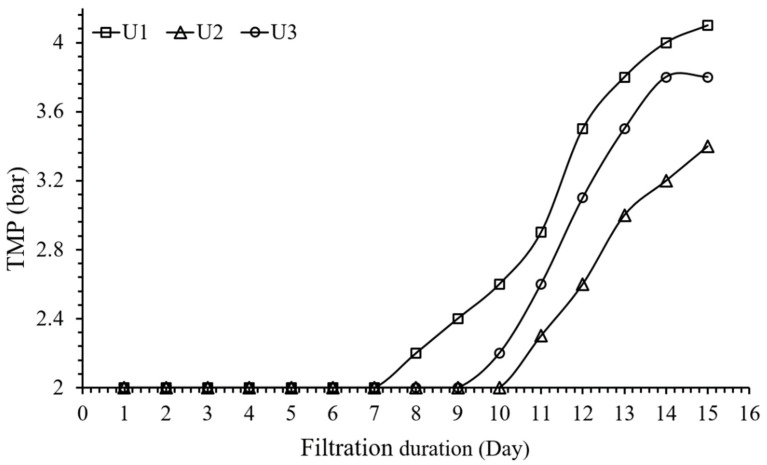
The permeate flux and salt rejection in RO operation for U1, U2, and U3, operated with control, CO_2_, and antifouling agent, respectively.

The operational performance of all the units was examined for 15 days and the process efficiency was identified based on the change in TMP. All units were operated at 2 bar initial TMP to achieve 56% recovery. The control unit (U1) indicated successful operation for the initial seven days, as shown in Figure 2. Later, a gradual rise in TMP was noticed during the extended period of filtration. On the 15th day, the TMP of U1 reached 4.1 bar, twice higher than the initial TMP. The TMP increase exhibits the resistance generated by the polarization layer, ionic deposition, or development of the cake layer on the surface of the polyamide spiral-wound membrane. A noticeable rise in the rate of ionic deposition within the RO unit contributed significantly to the rise in TMP. It is a known fact that a higher pressure is needed to maintain the constant permeate flux [17]. Nearly a 90% rise in initial TMP was observed for U3, which operated with the antifouling agent MDC-220. The closing value of TMP for U3 was 3.8 bar. Conversely, U2 exhibited higher stability in terms of maintaining TMP during the period of filtration. During the first 10 days, no change in TMP was observed for U2. TMP marginally increased in the following days, reaching a maximum value of 3.4 bar when the plant was shut down. Based on the operational performances, the trend for operational proficiency of all the units can be drawn as U1 > U3 > U2. The results confirmed that the scale formation and development of the cake layer on the membrane’s surface were under control when the feed water was conditioned with CO_2_.

As all the units were operated in constant flux mode, an increase in the duration of filtration and, subsequently, ionic precipitation, seriously influenced the TMP. The rise in TMP is assumed to be the result of the consistent deposition of multivalent ions onto the surface of the membrane. In addition, the contribution of concentration polarization to reducing the process efficacy after several cycles of filtration cannot be ignored. Higher concentration polarization in the neighborhood of membrane surfaces triggers the excessive accumulation of solute concentration at the membrane surface, which conspicuously aids the progression of a cake layer [17]. As concentration polarization layers principally appear in conjunction with the surfaces of membranes, the lengths of membrane feed channels impact the solute concentration. The higher velocity reduces concentration polarization due to improved mass transfer and reduced yield [18]. U1 and U3 displayed much lower productivity in terms of maintaining TMP during the entire operation. The TMP change at the initial stage of filtration does not signify extensive scaling, which would impede long-term operation. However, after 7 days of operation, both units showed a swift rise in TMP, an indication of rapid scaling. The excessive increase in TMP could be a consequence of foulants entrenched in the membrane feed spacers [19]. The rise in TMP profile corresponded with the formation of the cake layer, which abruptly enhanced the resistance to water transport through the RO membrane. It can be specified that the dominancy of concentration polarization in the neighborhood of membrane surfaces may escalate osmotic pressure. Particularly, the solubility limits of multivalent ions (such as Mg^2+^, Ca^2+^, etc.) can pile up and the subsequent precipitation of ions can leave serious impressions on the mass transport phenomena [20].

In addition, to reduce the pH of synthetic wastewater, dissolved CO_2_ molecules are known to play a role in the demolition of deposited layers of scale on the surfaces of RO membranes [16]. The voids found within membrane surfaces can be anticipated to be CO_2_ nucleation vicinities, thus lowering the free interfacial energy essential for CO_2_ nucleation. Moreover, the fouling layer evolved onto the membrane surface may also serve as a substrate for CO_2_ nucleation [21]. The dissolved CO_2_ molecules would, in all probability, travel into the apertures inside the scale layer, prompting the nucleation, progression, and extrication of CO_2_ molecules. Likewise, the surfeit presence of voids might be prevised in a porous cake layer compared to a thick gel layer, therefore encouraging CO_2_ nucleation and the resultant preservation of permeate flux. The fouled RO membranes were thoroughly washed with reported cleaning protocols [5]. The contact time between the cleaning solution and membranes was altered within a range of 10–60 min. The effect of contact time on the efficiency of CP is examined and discussed in Section 3.4.

### 3.2. Visual Examination of the Fouled RO Membranes

**Figure 3:**
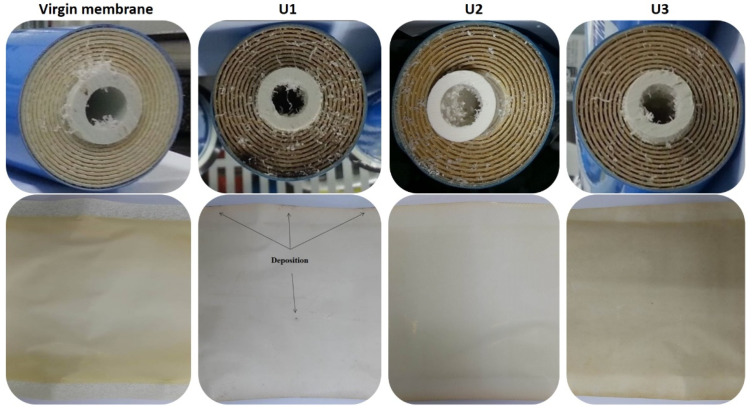
The visual examination of membranes ((**top**): inlet cross-section; (**bottom**): unfolded membrane surfaces) used in this study.

In the visual examination of the spiral-wound membrane element, outer wrap, brine seal, pressure vessel, and perforated product tube, no physical damage was found, which confirmed the nonexistence of any pressure-induced damage. The membrane elements were unfolded and inspected layer by layer. Figure 3 shows images of the inlet area and membrane sheets of the virgin and fouled membranes. The color of the used membranes faded due to continuous filtration. The inlet zone of the control membrane element was found with black deposits. The blackish marks found on the feed channel spacers of the control membrane showed severe deformation. Earlier studies also found similar results during RO operation, due to a higher TMP or accumulation of biofoulants [22]. Considering the operational data, it can be stated that after several filtration cycles, the rising TMP led to the deformation of the control membrane element. The application of a phosphonate-based antifouling agent can also increase the potential of biofouling. Antifouling agents of the phosphonates group can enhance the concentration of phosphate in feed water. Phosphonate degradation and by-product production can also lead to serious challenges for RO units [23]. On the whole, U2 and U3 did not show any deformation, biofouling, or specific odor at the inlet zone of the membrane elements. A fine layer of scale was found on both sides of the membrane sheets. The scale layer was spread over the surfaces of the membranes and there was no major difference between the influent and permeate zones of the membranes. Moreover, no channeling effect was identified on the surface of the membranes.

### 3.3. Membrane Surface Analysis

**Figure 4:**
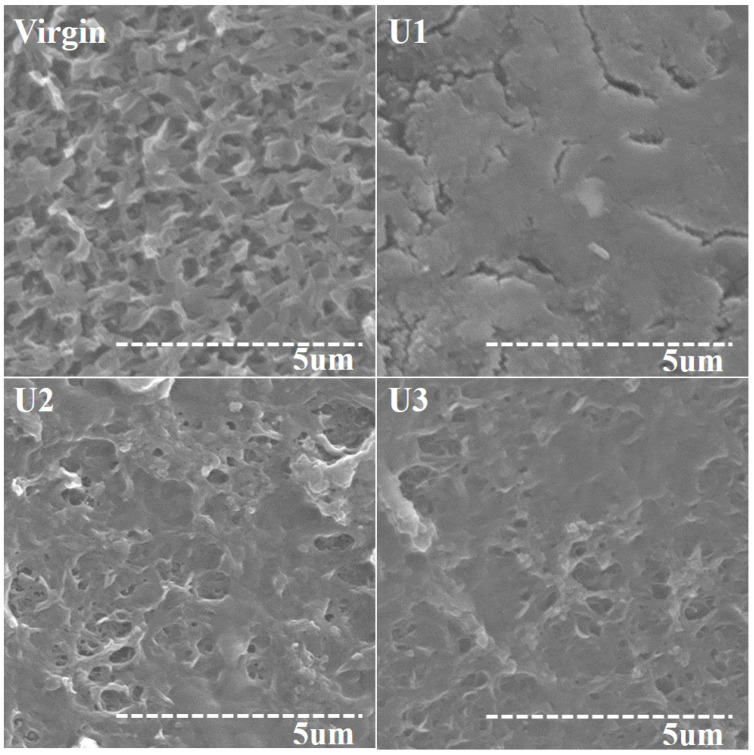
The surface morphology of virgin and fouled membranes.

**Figure 5:**
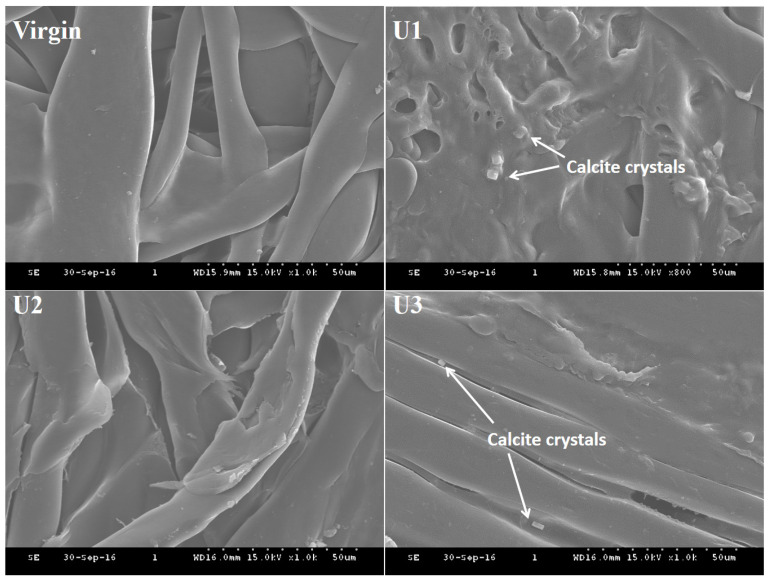
The higher-magnified SEM images of membrane surfaces.

Upon the shutdown of all the units, the membrane surfaces were investigated in order to identify the nature of ionic deposition. The morphology of the scale deposits and their elemental composition were identified using SEM and EDS, respectively. The surface analysis of the virgin and used membranes exhibited a prominent difference in morphology of scale (Figure 4). The surface of the U1 membrane was fully covered by a scale layer that caused TMP to increase over the filtration period. When the surfaces of the membranes were examined with a higher magnification (Figure 5), well-developed rhombohedral calcite crystals were observed in the fouling layer that developed on the surface of the U1 and U3 membranes. The fibrous structures found in the scale layer indicate the assembly of calcium carbonate crystals and other inorganic constituents [20]. The shape and structure of the scale deposits were similar to the reported morphology of calcite and aragonite [24]. The mud-like layer is indicative of an excess deposition of salts containing sodium and potassium [25]. It is a known fact that the rate of fouling deposition and the shape and structure of foulants significantly affect the fouling resistance and process efficiency in filtration systems [26].

Based on the morphological inspection, the trend for scale load on the membrane surfaces from all the units can be drawn as U1 membrane > U3 membrane > U2 membrane. Most of the surface area of the U2 membrane was either free of foulants or somewhat exposed to foulants. This confirms that CO_2_ introduction in the influent of membrane-based water treatment systems can significantly reduce membrane fouling. The composition of feed water also affects crystal morphology.

**Figure 6:**
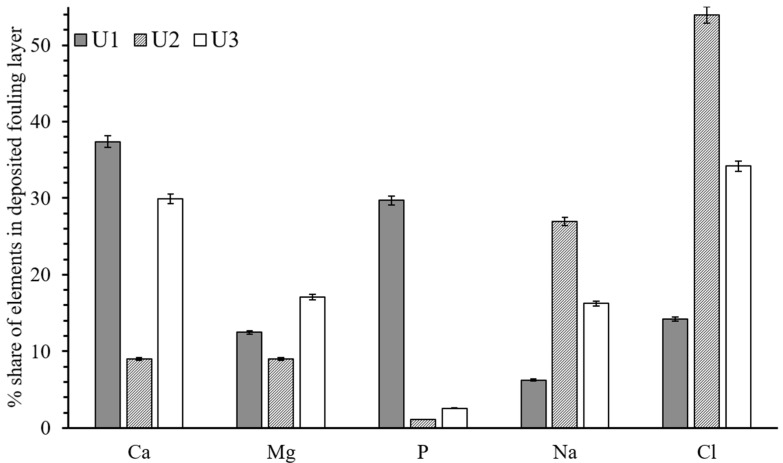
The elemental composition of the accumulated scale layer on membrane surface.

The elemental analysis of the fouling layer was conducted to understand the chemical composition of the foulants. The virgin polyamide membrane contains the major fraction of carbon, nitrogen, oxygen, and sulfur in its structure. Hence, these elements were ignored when the elemental analysis was conducted. The accumulated foulants were made up of different inorganic ions, including calcium, magnesium, phosphorous, sodium, and chloride. The membrane separated from U1 carried a major share of the calcium and magnesium. The scale formed on the surface of the U1 membrane was found to have many inorganic elements, including calcium, magnesium, phosphorus, sodium, and chloride that were at averages of 37.38%, 12.46%, 29.69%, 6.23%, and 14.22% by weight percentages, respectively (Figure 6). The major weight share of calcium shows that the surface of the membrane was mainly covered by CaCO_3_ [27]. The high weight percentage of phosphorus and calcium might be a sign of calcium phosphate deposition [28]. A relatively smaller weight share of calcium was identified in the fouling layer of the membrane separated from U3. In the case of the U2 membrane, the weight share of calcium and magnesium was less than 9%, whereas the major share was from sodium and chloride, which is possibly due to the excessive presence of these two ions in the feed water. Based on the surface examination of the fouled membranes, it can be established that the operation of membrane-based water treatment plants with CO_2_ is highly effective for scale inhibition.

### 3.4. Evaluation of CP

**Figure 7:**
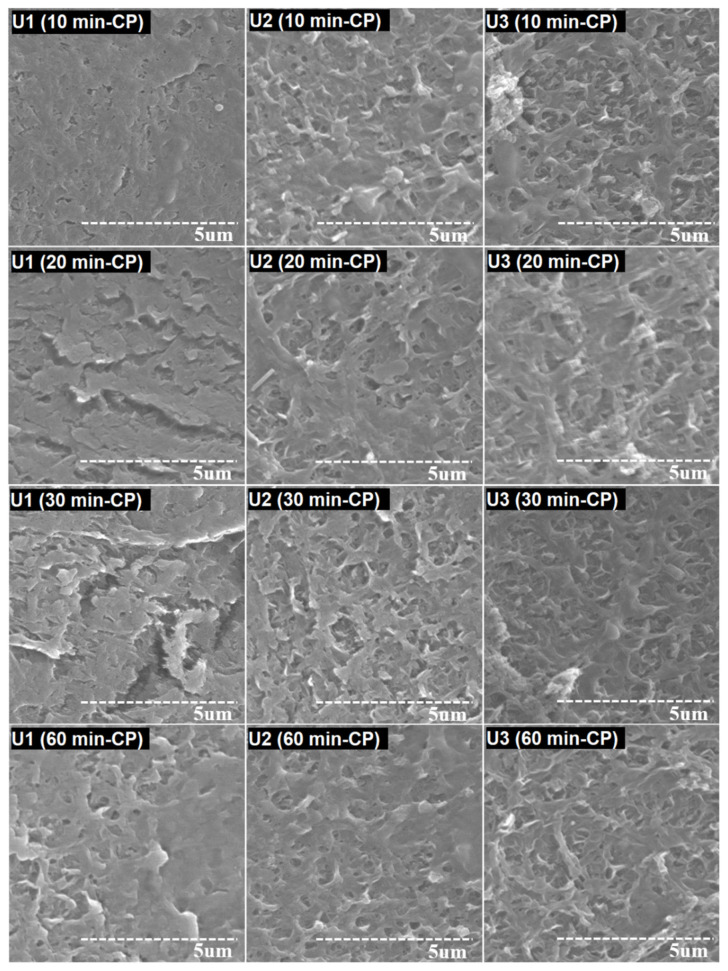
SEM images of RO membranes after 10, 20, 30, and 60 min of chemical cleaning.

The fouled membranes were cleaned using 0.1% NaOH, 0.2% HCl, and 1% EDTA solutions. The membrane specimens were obtained from different locations on the membrane element. The membrane samples were kept in cleaning solutions for a certain time, and then the surface examination was conducted. Cleaning was conducted for different sets of contact times, ranging from 10 to 60 min. Initially, cleaning was carried out for 10 min. The membrane surface of U1 was found to be highly spoiled and fully covered by a slurry-like precipitation. The ineffectiveness of chemical cleaning on the membrane surface of U1 indicated the highly compact and crowded crystallization of small particles on the surface of the membrane. A number of CaCO_3_ deposits were observed on the membrane surface of U3, while little deposition was observed on the membrane surface of U2 (Figure 7).

The 10 min CP was found to have successfully removed most of the deposits from the surface of the U2 and U3 membranes. A more enhanced cleaning efficiency was achieved with a 20 min contact time. A slurry-like film with breaks was observed on the surface of the U1 membrane, which showed the slight effect of chemical cleaning. The U2 and U3 membranes were found with a minor existence of foulants. The calcium carbonate structures were observed on the membrane surface of U3, which shows that the membrane was carrying a significant amount of scale prior to cleaning practices.

All the depositions from the surface of U2 were successfully removed by chemical cleaning for 30 min. The membrane surface of U1 was partially covered with a sludge-like layer (Figure 7). Moreover, calcite and aragonite crystals were also observed on the surface of the U1 membrane. A fibrous structure of aragonite was also observed on the surface of the U3 membrane. No scale deposits were observed on the surfaces of the membranes from U2 and U3 after chemical cleaning for an hour. The surface of the U1 membrane was covered with an irregular, slightly sludge-like layer, alongside calcite and aragonite crystals. It seems that the successful removal of depositions from membrane surfaces can be achieved by extending the duration of chemical cleaning.

**Figure 8:**
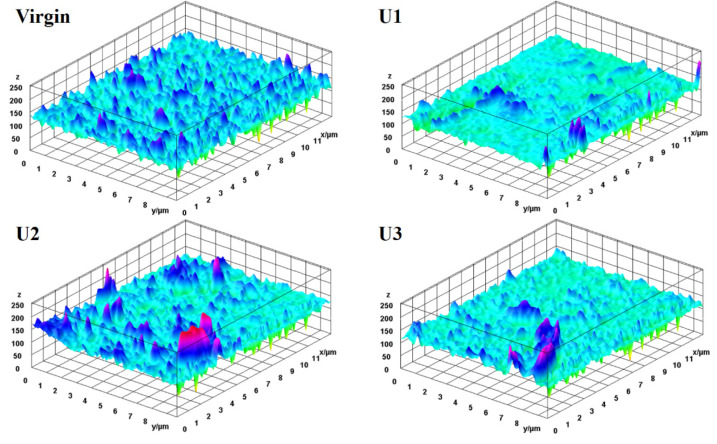
Topographical images of membranes after operation.

**Figure 9:**
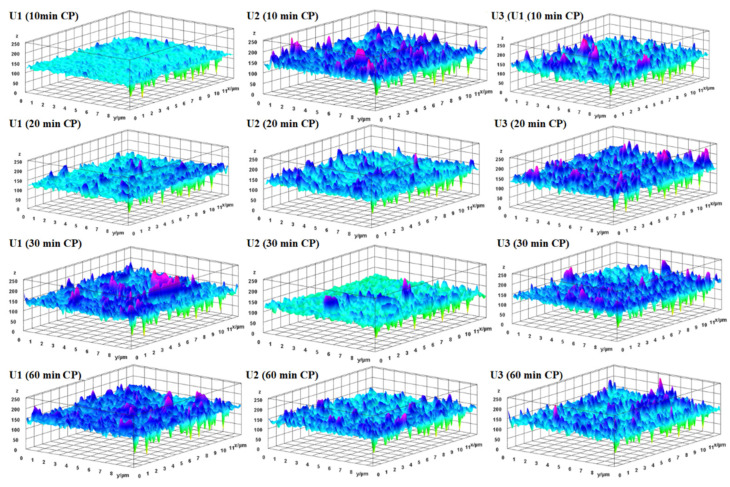
Topographical images of membranes after cleaning for different durations.

The surface roughness of the membranes was calculated based on the topographical images presented in Figure 8. The normal ridge–valley structure was found on the virgin membrane. The root mean square (RMS) of the virgin membrane was determined to be 131 nm, while it was changed to 124.3, 133.1, and 127.4 for U1, U2, and U3, respectively. The surfaces of the membranes became smoother due to the development of fouling layers. The reduced surface roughness found after the filtration cycle may be owing to the deposition of inorganic ions in the valley region. The ionic deposition in ridge and valley zones behaves differently depending on the nature of the foulants and the surface chemistry [29]. The RMS values were improved after chemical cleaning for different durations (Figure 9, Table 2), which shows the effectiveness of CP. In addition, chemical cleaning improved the surface roughness.

## 4. Conclusions

This study was designed to examine the efficiency of CO_2_ for the inhibition of inorganic scale growth in membrane-based water and wastewater treatment systems, and the effectiveness of cleaning protocols for the restoration of fouled membranes. CO_2_ was introduced into the feed water of the RO unit to decrease the solution pH and to control the fouling potential of the system. Three RO units were operated with a control, CO_2_, and a chemically synthesized antifouling agent. The operational performance of all the units was compared in terms of change in TMP and surface morphology. The efficiency trend for all the units was found as U1 > U3 > U2. The outcomes of this study confirmed that the fouling growth and development of the cake layer on the surface of the membrane was successfully inhibited when the feed water was conditioned with CO_2_. The results obtained from the morphological examination of the used membranes agreed with the operational data. The excess amount of scale identified on the surface of the control membrane was difficult to completely remove with chemical cleaning, even with a 60 min contact time. The U2 membrane showed a successful restoration of the membrane surface with 20–30 min cleaning. The surface roughness of the virgin membrane was decreased after the operation of the membranes; however, it was reinstated after chemical cleaning.

## Figures and Tables

**Table 1:** Specification of membranes used in this study.

Parameter	Analytical Result
Manufacturer	HYUNDAI Wacortec, Korea
Membrane material	Polyamide
Membrane configuration	Spiral-wound thin-film composite
Membrane area	0.4 m^2^
MWCO	~100 Da.
Permeate flowrate	50 GPD
Maximum operating temperature	113 °F
Maximum operating pressure	8.6 bar
Maximum feed flow rate	7.6 L/min
pH operating range	3–10

**Table 2:** The RMS values (nm) calculated based on the topographic images of the membranes after filtration operation and chemical cleaning.

Membrane	After Operation	After 10 min CP	After 20 min CP	After 30 min CP	After 60 min CP
U1	124.3	127.7	132.9	143.9	147.7
U2	133.1	142.9	135.0	142.0	137.1
U3	127.4	133.0	142.9	120.9	134.9

## Data Availability

The data presented in this study are available in the article.

## References

[1] Shen D., Shen D. (2021). Climatic Change and Water Resources. Water Resources Management of the People’s Republic of China.

[2] Ennaceri H., Fischer K., Schulze A., Moheimani N.R. (2022). Membrane Fouling Control for Sustainable Microalgal Biodiesel Production: A Review. Renew. Sustain. Energy Rev..

[3] Kashif A., Rehman R., Fuwad A., Shahid M.K., Dayarathne H.N.P., Jamal A., Aftab M.N., Mainali B., Choi Y. (2022). Current Advances in the Classification, Production, Properties and Applications of Microbial Biosurfactants—A Critical Review. Adv. Colloid Interface Sci..

[4] Chen G.-Q., Wu Y.-H., Fang P.-S., Bai Y., Chen Z., Xu Y.-Q., Wang Y.-H., Tong X., Luo L.-W., Wang H.-B. (2022). Performance of Different Pretreatment Methods on Alleviating Reverse Osmosis Membrane Fouling Caused by Soluble Microbial Products. J. Memb. Sci..

[5] Shahid M.K., Pyo M., Choi Y. (2017). Carbonate Scale Reduction in Reverse Osmosis Membrane by CO_2_ in Wastewater Reclamation. Membr. Water Treat..

[6] Amiria A., Al-Rawajfehb A., Brewera C.E. (2018). Simulation of Small-Scale Thermal Water Desalination Using Biomass Energy. Desalin. Water Treat..

[7] Al-Maabreh A.M., Al-Rawajfeh A.E., Alshamaileh E., Al-Bazedi G.A. (2019). Mitigation of Scale Problem in the Pumped Disi Water to Amman, Jordan. Environ. Prot. Eng..

[8] Dayarathne H.N.P., Choi J., Jang A. (2017). Enhancement of Cleaning-in-Place (CIP) of a Reverse Osmosis Desalination Process with Air Micro-Nano Bubbles. Desalination.

[9] Dayarathne H.N.P., Jeong S., Jang A. (2019). Chemical-Free Scale Inhibition Method for Seawater Reverse Osmosis Membrane Process: Air Micro-Nano Bubbles. Desalination.

[10] Shirazi S., Lin C.J., Chen D. (2010). Inorganic Fouling of Pressure-Driven Membrane Processes—A Critical Review. Desalination.

[11] Aslam M., Yang P., Lee P.H., Kim J. (2018). Novel Staged Anaerobic Fluidized Bed Ceramic Membrane Bioreactor: Energy Reduction, Fouling Control and Microbial Characterization. J. Memb. Sci..

[12] Chen G.-Q., Wu Y.-H., Tan Y.-J., Chen Z., Tong X., Bai Y., Luo L.-W., Wang H.-B., Xu Y.-Q., Zhang Z.-W. (2022). Pretreatment for Alleviation of RO Membrane Fouling in Dyeing Wastewater Reclamation. Chemosphere.

[13] Unal B.O. (2022). Membrane Autopsy Study to Characterize Fouling Type of RO Membrane Used in an Industrial Zone Wastewater Reuse Plant. Desalination.

[14] Rolf J., Cao T., Huang X., Boo C., Li Q., Elimelech M. (2022). Inorganic Scaling in Membrane Desalination: Models, Mechanisms, and Characterization Methods. Environ. Sci. Technol..

[15] Ngene I.S., Lammertink R.G.H., Kemperman A.J.B., Van De Ven W.J.C., Wessels L.P., Wessling M., Van Der Meer W.G.J. (2010). CO_2_ Nucleation in Membrane Spacer Channels Remove Biofilms and Fouling Deposits. Ind. Eng. Chem. Res..

[16] Shahid M.K., Choi Y. (2021). Sustainable Membrane-Based Wastewater Reclamation Employing CO_2_ to Impede an Ionic Precipitation and Consequent Scale Progression onto the Membrane Surfaces. Membranes.

[17] Cai Y.-H., Galili N., Gelman Y., Herzberg M., Gilron J. (2021). Evaluating the Impact of Pretreatment Processes on Fouling of Reverse Osmosis Membrane by Secondary Wastewater. J. Memb. Sci..

[18] Jung O., Saravia F., Wagner M., Heißler S., Horn H. (2019). Quantifying Concentration Polarization—Raman Microspectroscopy for In-Situ Measurement in a Flat Sheet Cross-Flow Nanofiltration Membrane Unit. Sci. Rep..

[19] Rahardianto A., Gu H., Khan B.M., Plumlee M.H. (2020). Real-Time Reverse Osmosis Monitoring for Antiscalant Dose Selection in Advanced Treatment of Wastewater. AWWA Water Sci..

[20] Shahid M.K., Choi Y.-G. (2017). The Comparative Study for Scale Inhibition on Surface of RO Membranes in Wastewater Reclamation: CO_2_ Purging versus Three Different Antiscalants. J. Memb. Sci..

[21] Alpatova A., Qamar A., Al-Ghamdi M., Lee J., Ghaffour N. (2020). Effective Membrane Backwash with Carbon Dioxide under Severe Fouling and Operation Conditions. J. Memb. Sci..

[22] Shahid M.K., Pyo M., Choi Y.-G. (2017). The Operation of Reverse Osmosis System with CO_2_ as a Scale Inhibitor: A Study on Operational Behavior and Membrane Morphology. Desalination.

[23] Vrouwenvelder J.S., Beyer F., Dahmani K., Hasan N., Galjaard G., Kruithof J.C., Loosdrecht M.C.M. (2010). Van Phosphate Limitation to Control Biofouling. Water Res..

[24] Cai Y.-H., Burkhardt C.J., Schäfer A.I. (2021). Renewable Energy Powered Membrane Technology: Impact of Osmotic Backwash on Scaling during Solar Irradiance Fluctuation. J. Memb. Sci..

[25] Guillen-Burrieza E., Thomas R., Mansoor B., Johnson D., Hilal N., Arafat H. (2013). Effect of Dry-out on the Fouling of PVDF and PTFE Membranes under Conditions Simulating Intermittent Seawater Membrane Distillation (SWMD). J. Memb. Sci..

[26] Lin D., Bai L., Xu D., Zhang H., Guo T., Li G., Liang H. (2021). Effects of Oxidation on Humic-Acid-Enhanced Gypsum Scaling in Different Nanofiltration Phases: Performance, Mechanisms and Prediction by Differential Log-Transformed Absorbance Spectroscopy. Water Res..

[27] Farhat S., Bali M., Kamel F. (2018). Membrane Autopsy to Provide Solutions to Operational Problems of Jerba Brackish Water Desalination Plant. Desalination.

[28] Rathinam K., Modi A., Schwahn D., Oren Y., Kasher R. (2022). Surface Grafting with Diverse Charged Chemical Groups Mitigates Calcium Phosphate Scaling on Reverse Osmosis Membranes during Municipal Wastewater Desalination. J. Memb. Sci..

[29] Wang M., Wang J., Jiang J. (2022). Membrane Fouling: Microscopic Insights into the Effects of Surface Chemistry and Roughness. Adv. Theory Simul..

